# Persistent presumed allergic fungal rhinosinusitis during tezepelumab therapy with improvement after revision surgery: a case report

**DOI:** 10.3389/falgy.2026.1944701

**Published:** 2026-08-31

**Authors:** Bashayer Saad Hamoud Alsultan, Aasem Gharamah Alshehri, Leen Abdulmohsin Sarhan, Ghadah Saad H. Alsultan, Yahya Shabi

**Affiliations:** 1 Department of Internal Medicine, Aseer Central Hospital, Abha, Saudi Arabia; 2 College of Medicine, King Khalid University, Abha, Saudi Arabia; 3 Department of Microbiology and Clinical Parasitology, College of Medicine, King Khalid University, Abha, Saudi Arabia

**Keywords:** allergic fungal rhinosinusitis, anti-TSLP, biologic therapy, case report, chronic rhinosinusitis with nasal polyps, mucocele, tezepelumab

## Abstract

Allergic fungal rhinosinusitis (AFRS) is a clinically distinct subset of type 2 chronic rhinosinusitis with nasal polyps (CRSwNP), characterized by markedly elevated total IgE, fungal sensitization, and expansile disease with bony remodeling. Definitive diagnosis requires all five Bent and Kuhn criteria, including histopathological demonstration of eosinophilic mucin without tissue invasion and a positive fungal stain. Tezepelumab, a monoclonal antibody against thymic stromal lymphopoietin (anti-TSLP), has phase 3 evidence in severe CRSwNP but has not been evaluated specifically in AFRS. We report a 32-year-old woman with an AFRS-like CRSwNP phenotype, clinically and radiologically suspected but not histologically confirmed (total serum IgE 1,330 IU/mL; fungal polysensitization to Aspergillus, Alternaria, Penicillium and Candida) and severe asthma. During 11 months of tezepelumab prescribed for severe asthma rather than initiated for sinonasal disease, sinonasal disease was not controlled: the 22-item Sino-Nasal Outcome Test (SNOT-22) score remained severe (85→89/110) and imaging showed interval progression of maxillary disease, with persistence of a left posterior ethmoidal mucocele encroaching on the optic canal and orbit that had already been present on pre-biologic imaging. Revision endoscopic sinus surgery (FESS) was required; two months postoperatively the SNOT-22 improved markedly to 48/110 and asthma control improved partially (Asthma Control Test [ACT] 8→15/25). The temporal pattern is compatible with improvement following surgery rather than anti-TSLP therapy, although perioperative corticosteroids, continued medical treatment, changes in adherence and the natural course of disease may also have contributed. A single, uncontrolled observation with only two months of postoperative follow-up cannot establish that TSLP blockade is ineffective in AFRS; it does illustrate that fixed structural disease may not be addressed by biologic therapy alone. Dedicated prospective studies of anti-TSLP therapy in histologically confirmed AFRS are needed.

## Introduction

CRSwNP affects approximately 1%–4% of adults and is most often driven by type 2 mucosal inflammation involving IL-4, IL-5 and IL-13 (1, 2). AFRS is a clinically distinct subset defined by the Bent and Kuhn criteria, type I hypersensitivity to fungi, nasal polyposis, characteristic CT findings, eosinophilic mucin without tissue invasion, and a positive fungal stain of surgical contents, all five being required for a definitive diagnosis (3, 4). Where tissue is not available, the diagnosis can only be regarded as presumed or clinically suspected, and this distinction is maintained throughout the present report. It typically shows markedly elevated total IgE, fungal polysensitization, and expansile disease with bony remodeling that may extend toward the orbit and skull base (4, 5). Paranasal sinus mucoceles are slow-growing, epithelium-lined cystic lesions that follow chronic ostial obstruction, favor the frontal and ethmoid sinuses, and can cause proptosis, diplopia and visual impairment through orbital compression (6, 7).

Tezepelumab reduced nasal-polyp burden, symptom and radiological scores, systemic corticosteroid use and the need for surgery in the phase 3 WAYPOINT trial (8), and U.S. prescribing information now includes an add-on maintenance indication for inadequately controlled CRSwNP (9, 10); it also has established efficacy in severe asthma (11). Dupilumab (anti-IL-4 receptor alpha) has a broad evidence base across upper- and lower-airway type 2 disease (12, 13). EPOS/EUFOREA guidance recommends endotype-aware patient selection and response assessment at approximately six months (14), but high-quality evidence specific to AFRS remains limited to case reports and small series (5, 15). We describe an AFRS-like, clinically suspected CRSwNP phenotype that progressed during tezepelumab therapy prescribed for coexisting severe asthma.

## Case description

A 32-year-old woman presented with long-standing CRSwNP, persistent nasal obstruction, anosmia and purulent nasal discharge despite medical and surgical therapy having undergone primary FESS approximately six years earlier with subsequent progressive polyp recurrence. Before biologic therapy and revision surgery she had severe patient-reported burden (22-item Sino-Nasal Outcome Test [SNOT-22] 85/110) and very poorly controlled, difficult-to-treat asthma (Asthma Control Test [ACT] 5/25), with frequent emergency-department attendances (approximately two to three per month by self-report) and intermittent controller non-adherence. She had also not implemented non-pharmacological environmental measures to reduce fungal exposure. She reported food-related anaphylactic reactions (attributed to chicken or chocolate) previously treated with intramuscular adrenaline and corticosteroids; a formal allergy work-up with a written anaphylaxis action plan was recommended.

### Concomitant medical therapy

Because interpretation of the observed response depends on background treatment, concomitant therapy before, during and after tezepelumab is summarized here and in Table 1. Before biologic therapy the patient was prescribed intranasal corticosteroid (mometasone) spray twice daily and as needed, with intermittent adherence, and also high-volume saline nasal irrigation was used. She received intermittent short courses (5 days per episode) of systemic corticosteroids (prednisolone) for asthma exacerbations and sinonasal symptoms. No systemic or topical antifungal therapy was administered at any point. Asthma treatment consisted of inhaled corticosteroid/long-acting beta-agonist combination therapy, budesonide/formoterol then switched to fluticasone furoate/vilanterol for convenience, continued unchanged throughout.

**Table 1:** Clinical timeline (CARE guideline), with concomitant treatment and outcome scores reported at each time point.

Date/period	Clinical event	Treatment in place	SNOT-22/ACT
∼2020	Primary FESS for nasal polyposis; later recurrent CRSwNP.	Intranasal corticosteroid; intermittent adherence	Not recorded
28 Jan 2025	Brain MRI: left posterior ethmoid expansion with eroded septa encroaching on the optic canal and orbit, reported as a likely mucocele with mucinous or fungal contents; partial empty sella (normal variant).	ICS/LABA; intranasal corticosteroid	Not recorded
Baseline (pre-biologic/pre-revision ESS)	Severe baseline patient-reported burden.	ICS/LABA; intranasal corticosteroid; intermittent systemic corticosteroid courses	SNOT-22 85/110; ACT 5/25
Apr 2025	Baseline eosinophils 0.10 × 10⁹/L; total serum IgE 1,330 IU/mL.	As above; no antifungal therapy at any time	–
∼May 2025	Tezepelumab 210 mg SC every 4 weeks initiated for severe asthma.	Tezepelumab added; all other therapy continued unchanged	–
22 Oct 2025	CT-PNS: interval progression of maxillary opacification with internal hyperdensity and ostiomeatal-complex remodeling; reported as suggestive of underlying fungal disease (Figure 1).	Tezepelumab; ICS/LABA; intranasal corticosteroid	–
Dec 2025	Specific IgE: class 4 D. pteronyssinus; class 2 Aspergillus, Alternaria, Penicillium, Candida.	Unchanged	–
Month 11 (pre-revision ESS)	Sinonasal disease persisted/worsened despite partial asthma-control improvement.	Tezepelumab (11 months); unchanged background therapy	SNOT-22 89/110; ACT 8/25
12 Apr 2026	Revision FESS: bilateral uncinectomy, wide maxillary antrostomy, anterior and posterior ethmoidectomy and wide sphenoidotomy with clearance of thick secretions and polypoid mucosa; skull base and lamina papyracea preserved. No specimen submitted for histopathology or fungal staining. Eosinophils 13 Apr 2026: 0.01–0.02 × 10⁹/L.	Perioperative corticosteroid and prophylactic antibiotic regimen	–
21 Apr 2026	Postoperative CT: regression of ethmoid opacity but mildly increased maxillary polypoid opacity; residual frontal and sphenoid disease; no significant overall interval change (Figure 2).	Tezepelumab continued; saline irrigation and intranasal corticosteroid resumed	–
2 months post-FESS (∼Jun 2026)	Marked improvement following surgery.	Tezepelumab; saline irrigation; intranasal corticosteroid	SNOT-22 48/110; ACT 15/25
Planned	Transition to dupilumab; structured follow-up at 1, 3, 6 and 12 months.	Dupilumab 300 mg SC every 2 weeks (planned)	To be recorded at each visit

Follow-up beyond two months after revision surgery is not yet available; durability of the observed improvement therefore remains unknown.

During tezepelumab therapy the intranasal corticosteroid, saline irrigation and inhaled controller regimen were continued without change, and systemic corticosteroids were given only for acute exacerbations. Perioperatively she received steroids again. Postoperative care comprised saline irrigation, resumption of intranasal corticosteroid and continued tezepelumab until the planned transition to dupilumab. The overlap of perioperative corticosteroids and continued topical therapy with the postoperative period is an important confounder for the observed symptomatic improvement and is addressed in the Discussion.

Laboratory and allergen-specific IgE findings are summarized in Table 2.

**Table 2:** Laboratory and allergen-specific IgE profile, with sampling dates (baseline April 2025, allergen-specific IgE December 2025, post-treatment April 2026).

Investigation	Result	Reference range	Comment
Total serum IgE	1,330 IU/mL	<100 IU/mL	Markedly elevated; supports presumed AFRS
Absolute eosinophils — baseline (Apr 2025)	0.10 × 10⁹/L	0.05–0.50 × 10⁹/L	Pre-/early biologic; last systemic corticosteroid course 1 day before sampling
Absolute eosinophils — post-treatment (Apr 2026)	0.01–0.02 × 10⁹/L	0.05–0.50 × 10⁹/L	Suppressed on anti-TSLP ± steroids; sampled 13 April 2026, one day after revision FESS and 2 days after the most recent systemic corticosteroid dose
Specific IgE — D. pteronyssinus (d1)	Class 4	Class 4 = intensity 46.49–82.49	House dust mite
Specific IgE — Aspergillus fumigatus (m3)	Class 2	Class 2 = intensity 11.49–23.49	Fungal
Specific IgE — Alternaria alternata (m6)	Class 2	Class 2 = intensity 11.49–23.49	Fungal
Specific IgE — Penicillium notatum (m1)	Class 2	Class 2 = intensity 11.49–23.49	Fungal
Specific IgE — Candida albicans (m5)	Class 2	Class 2 = intensity 11.49–23.49	Fungal
CCD marker	Class 0	Class 0 = intensity 0–5.49 (negative)	Excludes cross-reactive carbohydrate determinants

Reference ranges are those of the Aseer Central Hospital laboratory. Specific IgE was measured by semiquantitative immunoblot, reported in semiquantitative intensity units (0–255) and mapped to classes 0–6; class ≥ 1 indicates sensitization. These values are not equivalent to kUA/L and are not directly comparable with ImmunoCAP-based results. Systemic corticosteroid exposure at the time of each eosinophil measurement is indicated, since recent corticosteroid use suppresses the peripheral eosinophil count independently of biologic therapy.

#### Baseline imaging

**Figure 1:**
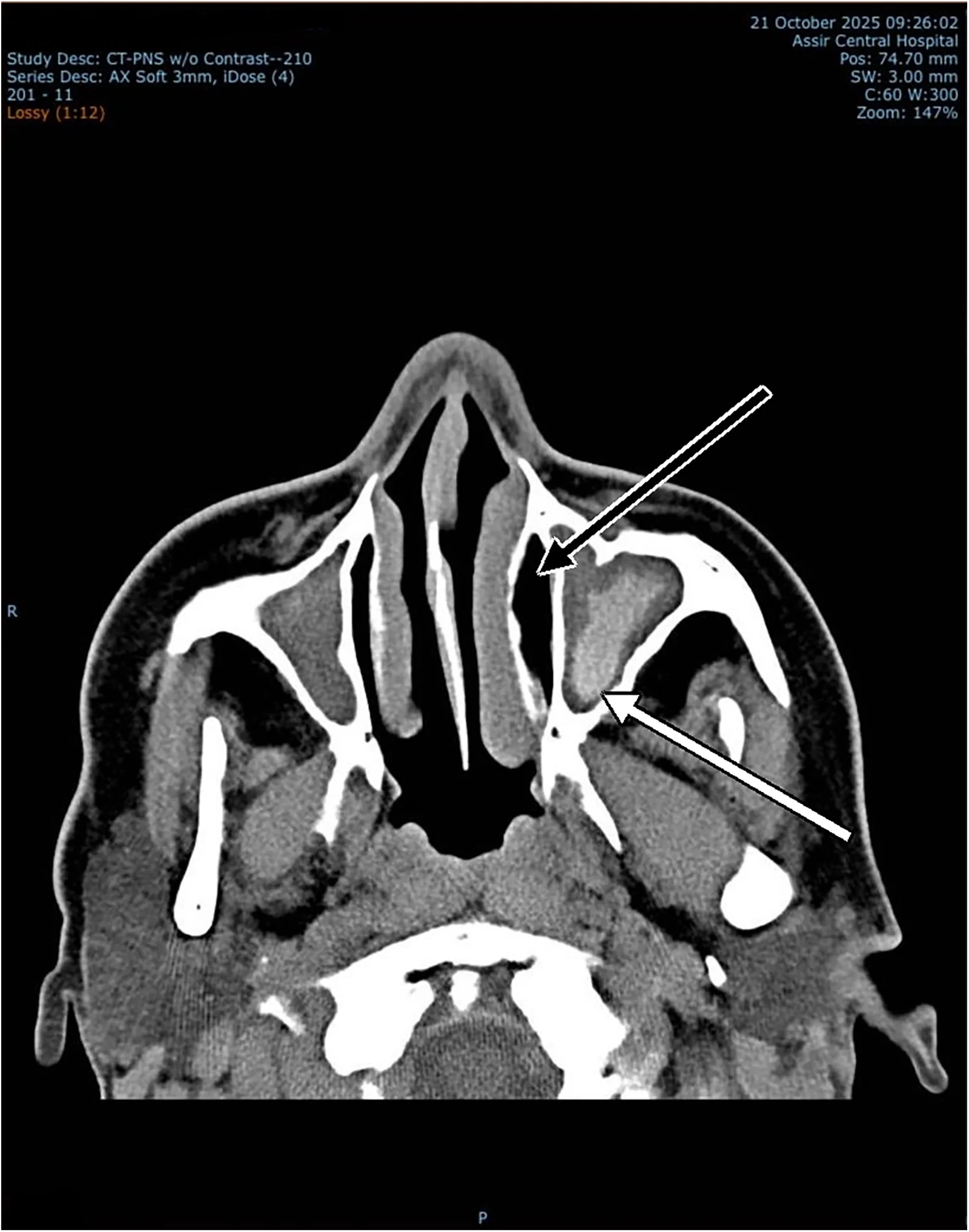
Axial CT of the paranasal sinuses (soft-tissue window), 22 October 2025, during anti-TSLP (tezepelumab) therapy, showing persistent sinonasal mucosal disease at the level of the maxillary sinuses and nasal cavity. Arrows indicate hyperdense material within the left maxillary sinus (white arrow) and remodeling of the left ostiomeatal complex (black arrow). Patient identifiers redacted.

Brain MRI (28 January 2025) showed ethmoid opacification with expansion and eroded septa of the left posterior cells, encroaching on the left optic canal and orbital cavity and reported as a likely mucocele; the T1-hyperintense, T2 iso-to-hypointense contents were attributed to mucin or fungal disease, and a partial empty sella was considered a normal variant. CT of the paranasal sinuses (22 October 2025) showed interval progression of maxillary opacification with internal hyperdensity and remodeling of the bilateral ostiomeatal complexes, more marked on the left, together with mild ethmoid expansion; the reporting radiologist considered the appearances suggestive of underlying fungal disease, with no orbital fat stranding and no evidence of lamina thinning (Figure 1).

## Disease course on tezepelumab and after surgery

**Figure 2:**
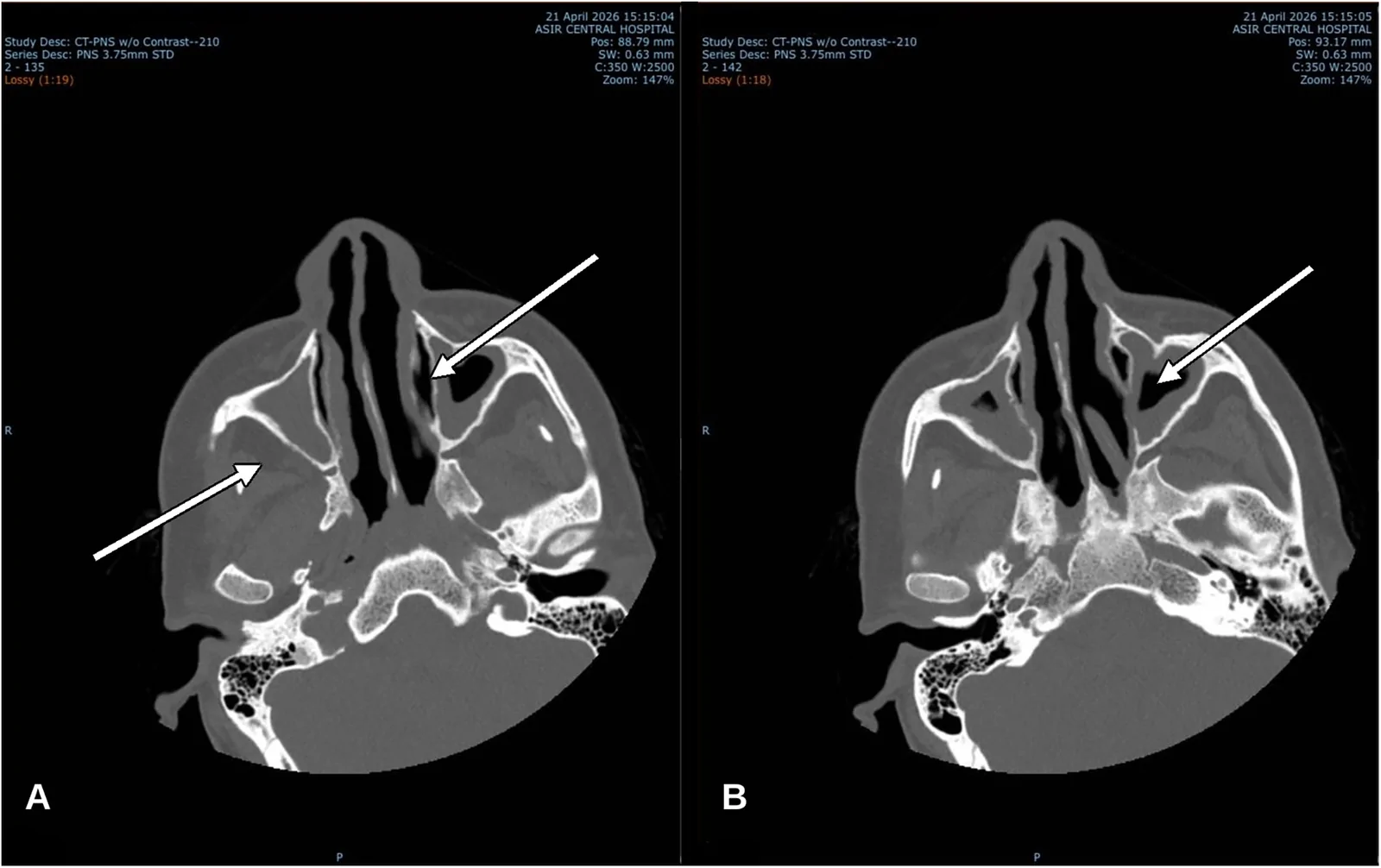
Axial CT of the paranasal sinuses (bone window), 21 April 2026, in the early period after revision FESS, showing post-surgical sinonasal cavities with residual mucosal disease and bony remodeling. **(A)** Maxillary/nasal level; the upper arrow indicates the widened left maxillary antrostomy and the lower arrow the residual opacification of the right maxillary sinus. **(B)** Ethmoid-sphenoid level; the arrow indicates the surgically opened left ethmoid cavity. Patient identifiers redacted.

Tezepelumab (210 mg subcutaneously every four weeks) was initiated for severe asthma, not for sinonasal disease; the sinonasal course was therefore observed rather than the target of a treatment decision. Over 11 months, nasal obstruction, anosmia and purulent discharge persisted without meaningful sinonasal improvement: the SNOT-22 score remained severe and rose from 85 to 89/110, while asthma control improved only modestly (ACT 5→8/25). The peripheral absolute eosinophil count was suppressed (0.01–0.02 × 10⁹/L), consistent with upstream epithelial-alarmin blockade and recent corticosteroid exposure and not excluding ongoing tissue eosinophilia. Because of persistent symptoms and radiological progression complicated by the radiologically presumed ethmoidal mucocele, revision FESS was performed (12 April 2026): bilateral uncinectomy and wide maxillary antrostomy, complete bilateral ethmoidectomy with removal of diseased ethmoid air cells and partitions, and wide bilateral sphenoidotomy with clearance of thick secretions and polypoid mucosa, with preservation of the skull base and lamina papyracea. Early postoperative CT (21 April 2026) showed regression of ethmoid opacity but mildly increased polypoid opacity in both maxillary sinuses, with residual total opacification of both frontal sinuses and bilateral sphenoid opacity; the reporting radiologist described no significant overall interval change from the October 2025 study (Figure 2).

Two months after surgery, patient-reported burden improved markedly: the SNOT-22 score fell from 89 to 48/110 (a 41-point reduction, well beyond the ≈9-point minimal clinically important difference), and the ACT score improved from 8 to 15/25 (above the ≈3-point MCID but still below the well-controlled threshold of 20). The improvement therefore coincided temporally with surgical clearance rather than with the preceding 11 months of anti-TSLP therapy, although it cannot be attributed to surgery alone, since perioperative corticosteroids, continued topical therapy, improved adherence after the intervention and the natural fluctuation of chronic rhinosinusitis may each have contributed. Histopathological examination of the sinus contents was not performed, and the diagnosis therefore remained clinical and radiological throughout; no fungal stain or culture of surgical material was obtained, so two of the five Bent and Kuhn criteria could not be assessed.

## Clinical timeline

## Discussion

The phenotype is AFRS-like but remains presumed rather than established. Markedly elevated total IgE (1,330 IU/mL), fungal polysensitization, recurrent polyposis, and hyperdense sinus contents with bony remodeling reported as suggestive of fungal disease each align with the Bent and Kuhn framework (3–5). However, because no histopathological examination of the sinus contents was undertaken, the two tissue-based criteria, eosinophilic mucin without tissue invasion and a positive fungal stain could not be assessed, and the diagnosis therefore remains clinical and presumptive rather than definitive (3, 4). Eosinophilic CRSwNP with fungal sensitization, and central compartment atopic disease, are plausible alternative labels for the same clinical picture, and the observations reported here would apply equally to those entities.

The radiologically presumed ethmoidal mucocele with optic-canal and orbital encroachment was the most consequential finding. Mucoceles follow ostial obstruction, favor the frontal and ethmoid sinuses, and threaten vision through orbital compression (6, 7). Revision FESS achieved mechanical clearance of the diseased ethmoid cells and partitions with preservation of the lamina papyracea and skull base, in keeping with standard surgical management; the lesion was not separately characterized in the operative record, and its designation therefore rests on imaging.

This point is central to the interpretation of the case. A mucocele, retained inspissated eosinophilic mucin and fixed bony remodeling of the ostiomeatal complex are mechanical rather than inflammatory endpoints. Once an epithelium-lined, expanding cavity has formed behind an obstructed ostium, no degree of upstream cytokine or alarmin blockade can restore ventilation or evacuate the retained secretions, and the same applies to allergic mucin impacted within a poorly ventilated ethmoid or sphenoid cavity, which acts as a persistent antigenic depot largely inaccessible to systemically delivered antibody. Biologic therapy in CRSwNP has been evaluated principally against polyp burden and mucosal inflammation, not against fixed structural obstruction, and the trials that established its efficacy did not enrol patients with expansile mucoceles threatening the orbit. The failure to improve during tezepelumab in this patient may therefore reflect the nature of the disease compartment as much as the pharmacological target, and it should not be read as evidence that TSLP blockade lacks anti-inflammatory activity in AFRS.

The WAYPOINT trial provides the strongest comparator context. In 408 adults with severe, uncontrolled CRSwNP, tezepelumab improved the nasal-polyp, nasal-congestion, SNOT-22 (mean difference −27.3), Lund-Mackay and total symptom scores and reduced decisions for surgery and systemic glucocorticoid use (8), although the trial enrolled a highly selected severe population, not all patients responded, and optimal positioning among surgery and other biologics remains unsettled (16). This patient's course contrasts with the average WAYPOINT response: her baseline SNOT-22 already exceeded the trial mean (85 vs. 68.7/110) and, after 11 months of tezepelumab, sinonasal disease worsened (89/110) with radiological progression requiring revision FESS. The subsequent 41-point SNOT-22 improvement coincided with surgery rather than with continued anti-TSLP therapy, and the biologic was in any case prescribed for asthma rather than selected for sinonasal disease; WAYPOINT was also neither designed nor powered to evaluate AFRS as a distinct subgroup. Allowing for the limitations below, this course is compatible with, but does not demonstrate, AFRS inflammation being only partly TSLP-dependent driven by intense fungal-antigen-driven Th2 and eosinophilic responses, very high local IgE and eosinophilic-mucin formation such that upstream alarmin blockade alone may be insufficient when fixed structural disease is present. Disease progression here was probably multifactorial: ongoing environmental fungal exposure, given that non-pharmacological avoidance measures were not implemented, together with intermittent adherence, may have contributed independently of the adequacy of anti-TSLP therapy. The temporal association does not establish causation either for the progression observed during tezepelumab or for the improvement observed after surgery.

Compared with the published experience of biologics in AFRS, this report differs in three respects. Most available data concern dupilumab: a regional case series of refractory AFRS reported symptomatic and radiological benefit (15), and scattered reports describe responses to omalizumab and to anti-IL-5 agents, generally in patients whose disease was mucosal and polypoid rather than expansile. Those reports also typically describe biologic therapy selected deliberately for sinonasal disease and continued for at least six months before response assessment, in line with EPOS/EUFOREA guidance (14). Here, by contrast, the biologic was an anti-alarmin agent prescribed for asthma, the sinonasal compartment contained a presumed mucocele with orbital encroachment at the time biologic therapy began, and the observation is one of non-response rather than of response. To our knowledge no previous report has described the sinonasal course of an AFRS-like phenotype during anti-TSLP therapy, which is the specific gap this case addresses; it is a hypothesis-generating observation about sequencing, whether structural disease should be cleared before, rather than during, biologic therapy and not a comparative statement about drug efficacy.

A clinical-radiological discordance was evident: symptoms improved markedly after surgery, whereas early postoperative CT showed regression of ethmoid opacity but mildly increased maxillary polypoid opacity and no significant overall interval change. Imaging obtained early after endoscopic sinus surgery reflects postoperative edema and retained secretions and is a poor time point for judging response, and symptom scores correlate only weakly with CT opacification in CRSwNP. The persistent maxillary polypoid disease nonetheless indicates ongoing type 2 inflammation despite anti-TSLP therapy, supporting a change in biologic class with radiological reassessment at a later interval.

Endotype-aware biologic selection is supported by international consensus and by the heterogeneous inflammatory signatures within polypoid CRS (2, 14, 17). After surgical clearance, transition to dupilumab (anti-IL-4 receptor alpha, 300 mg subcutaneously every two weeks) is planned, given dual upper- and lower-airway coverage and downstream blockade of IL-4 and IL-13 signaling (1, 17); dupilumab has shown favorable signals in a regional case series of refractory AFRS (15), although no head-to-head data exist and the choice is shared with the patient.

Limitations include the absence of histopathological confirmation, since no tissue specimen was submitted for histological analysis, which precludes definitive fulfillment of the Bent and Kuhn criteria; absence of formal endoscopic (modified Lund-Kennedy) and validated olfactory scoring at the key time points, low baseline and suppressed peripheral eosinophil counts that may underestimate tissue eosinophilia, and exacerbation data partly based on patient recall. The postoperative follow-up of only two months is a further and important limitation. AFRS and eosinophilic CRSwNP are chronically relapsing conditions in which recurrence after revision surgery is common and typically becomes apparent over 6 to 24 months; a two-month observation window therefore establishes an early symptomatic benefit but says nothing about the durability of that benefit, the recurrence rate, or the eventual need for further surgery. Early postoperative scores may also be favourably influenced by perioperative corticosteroids and by the immediate mechanical effect of cavity clearance. The conclusions drawn here should accordingly be regarded as provisional pending the planned 6- and 12-month assessments. SNOT-22 and ACT scores support the clinical interpretation but remain single-patient patient-reported outcomes, and a single uncontrolled case cannot support inference about the efficacy of any therapy. Spirometry, formal smell testing, Lund-Mackay CT scoring and record-verified exacerbations are being incorporated prospectively, and the report follows the CARE guideline (18).

## Conclusion

In a woman with severe asthma and an AFRS-like, clinically and radiologically presumed CRSwNP phenotype, sinonasal disease worsened (SNOT-22 85→89/110) during 11 months of anti-TSLP (tezepelumab) therapy prescribed for asthma and required revision FESS for a posterior ethmoidal mucocele with orbital encroachment; marked improvement then followed surgery (SNOT-22 89→48/110; ACT 8→15/25 at two months). Although anti-TSLP is now indicated for inadequately controlled CRSwNP, this phenotype was not associated with sinonasal improvement during anti-TSLP therapy, and the observed improvement followed surgery. Given the confounders of perioperative corticosteroids, continued medical therapy, adherence and natural course, and a follow-up of only two months, no causal conclusion about tezepelumab can be drawn from this single observation. Dedicated studies of anti-TSLP efficacy specifically in histologically confirmed AFRS are needed; endotype-aware biologic selection (for example, dupilumab) with structured response assessment is reasonable, and surgery remains essential where fixed structural disease such as a mucocele is present.

## Patient perspective

“For several years my blocked nose, loss of smell and constant nasal discharge affected my daily life, and my asthma was very hard to control despite treatment, with frequent visits to the emergency department. When I began the tezepelumab injections for my asthma, I hoped my nose and sinuses would also improve, but over nearly a year I noticed little change in the blockage or my sense of smell. After my sinus operation, however, I felt a clear difference: breathing through my nose became easier, the facial pressure eased, and my chest and asthma also improved, although not completely. I am grateful to my medical team for explaining each step, and I am continuing regular follow-up, including a planned change in my treatment, in the hope of further improvement.”

The patient's perspective was obtained directly from the patient at the two-month postoperative visit and is reported here in accordance with item 12 of the CARE checklist.

## Data Availability

The original contributions presented in the study are included in the article/Supplementary Material, further inquiries can be directed to the corresponding author.
